# Asiatic Acid Prevents Oxidative Stress and Apoptosis by Inhibiting the Translocation of α-Synuclein Into Mitochondria

**DOI:** 10.3389/fnins.2018.00431

**Published:** 2018-06-28

**Authors:** Hongqun Ding, Yuyun Xiong, Jing Sun, Chen Chen, Jing Gao, Huaxi Xu

**Affiliations:** ^1^Department of Clinical Laboratory Diagnostics, School of Medicine, Jiangsu University, Zhenjiang, China; ^2^Department of Clinical Laboratory, Affiliated Hospital of Jiangsu University, Zhenjiang, China; ^3^Department of Medicinal Pharmacy, School of Pharmacy, Jiangsu University, Zhenjiang, China

**Keywords:** α-synuclein, mitochondrion, apoptosis, asiatic acid, Parkinson disease

## Abstract

The association of α-synuclein (α-syn) with mitochondria occurs through interaction with mitochondrial complex I. Defects in this protein have been linked to the pathogenesis of Parkinson disease (PD). Overexpression of α-synuclein in cells has been suggested to cause elevations in mitochondrial oxidant radicals and structural and functional abnormalities in mitochondria. Asiatic acid (AA), a triterpenoid, is an antioxidant that is used for depression, and we have shown that pretreatment with AA can prevent PD-like damage, but its therapeutic effects in PD and mechanism remain unknown. In this study, we found that 0.5–2 mg AA/100 g diet significantly improves climbing ability in drosophila and extends their life-span—effects that we attributed to its antioxidant properties. AA also protected mitochondria against oxidative stress and apoptosis in a rotenone-induced cellular model. In an isolated mitochondria model, AA attenuated the decline in mitochondrial membrane potential that was induced by α-syn. Consequently, AA maintained membrane integrity and ATP production. Finally, we demonstrated that AA protects by blocking the translocation of α-syn into mitochondria. Our results suggest that mitochondria are crucial in PD and that AA is an excellent candidate for the prevention and therapy of this disease.

## Introduction

Parkinson disease (PD) is a progressive neurodegenerative disorder that is characterized by the preferential loss of dopaminergic neurons in the substantia nigra pars compacta (SNpc)(Moore et al., 2005) and the formation of intracytoplasmic protein aggregates, termed Lewy bodies, a major component of which is α-synuclein (α-syn) (Spillantini et al., 1997). Increased expression of α-syn has been hypothesized to cause familial and sporadic PD, culminating in a loss of nigrostriatal dopaminergic neurons and motor deficits (Feany and Bender, 2000; Rochet et al., 2004; Lee and Trojanowski, 2006; Jellinger, 2012).

Mitochondrial dysfunction has been implicated in pathogenesis of PD, in which α-syn is central. In α-syn-expressing dopaminergic neuronal cultures, α-syn can translocate to the mitochondria, interact with respiratory complex I, and interfere with mitochondrial function (Devi et al., 2008). Moreover, recombinant human α-syn leads to a dose-dependent loss of mitochondrial transmembrane potential and phosphorylation capacity when incubated with isolated rat brain mitochondria (Banerjee et al., 2010). Hoogerheide et al. (2017) found that α-syn molecules could be captured by a voltage-dependent anion channel (VDAC) using free energy considerations that allow α-synuclein translocation and retraction.

Rotenone, a mitochondrial complex I inhibitor, causes the loss of ATP and increases α-syn levels and oxidative stress (Sherer et al., 2002). Most *in vitro* models of PD are “acute” models that might fail to mimic exactly the characteristics of PD, a chronic neurodegenerative disease. Therefore, we have used a “chronic” model with a low dose (5 nM) of rotenone for 4 weeks in human neuroblastoma SH-SY5Y cells (Sherer et al., 2002). Sherer et al. (2002) concluded that this model can be used efficiently to screen anti-PD drugs.

Asiatic acid (AA) is a triterpene that is extracted from *Centella asiatica* (L.) Urban (Umbelliferae), which has been used widely as an antioxidant and anti-inflammatory herb in Ayurvedic medicine and traditional Chinese medicine. AA has neuroprotective properties in cell culture and animal systems (Mook-Jung et al., 1999; Lee et al., 2000, 2012; Krishnamurthy et al., 2009), protecting neurons from C2 ceramide-induced cell death by antagonizing mitochondria-dependent apoptosis (Zhang et al., 2012). We have also reported that AA has neuroprotective effects through mitochondrial pathways (Xiong et al., 2009a; Xu et al., 2012).

In this study, we found that AA protects against Parkinson-like injury in drosophila, SH-SY5Y cells, and isolated mitochondria. Notably, the mechanism is related to direct prevention of mitochondrial permeability transition pores (MPTPs) opening and inhibition of the translation of α-syn to mitochondria. These results confirm that AA is a candidate molecule for the prevention or therapy of PD.

## Materials and Methods

### Cells and Reagents

Human neuroblastoma SH-SY5Y cells were a gift from Dr. Zunji Ke, Institute for Nutritional Science, Chinese Academy of Sciences (Shanghai, China). Human recombinant wild-type α-syn from Escherichia coli was purchased from ProSpec-Tany TechnoGene Ltd. The purity was greater than 95% by RP-HPLC, according to the company. AA was obtained from Sigma (St. Louis, MO, United States); β-actin, α-syn, cytochrome C (Cyt C), peroxisome proliferator-activated re-ceptor gamma coactivator-1α (PGC-1α), BAX, and VDAC primary antibodies were obtained from Abcam (Cambridge, MA, United States); all secondary antibodies were obtained from Boster Biological Technology (Wuhan, China). All other reagents were acquired from commercial suppliers and were standard biochemical quality-grade.

### Drosophila Culture

Transgenic Drosophila α-syn was a gift from Dr. Liu Jiankang, Xi’an Jiaotong University. According to previous methods (Long et al., 2009), non-PD flies (UAS wild-type alpha-synuclein/+) and PD flies (Ddc-GAL4/+; UAS wild-type alpha-synuclein/+) were housed in bottles, on the bottoms of which was medium that contained agar, cornmeal, sucrose, water, dried yeast, and propionic acid, at 25°C with a 12-h light-dark cycle. PD drosophila were divided into five groups: PD, 0.5 mg AA/100 g medium, 1 mg AA/100 g medium, 1 mg AA/100 g medium, and 2 mg alpha lipoic acid (LA)/100 g medium.

### Climbing Assay and Life Span Observation

The climbing assay was performed as described (Feany and Bender, 2000; Pendleton et al., 2002). Briefly, every 230 flies were placed into one group, and every 10 flies were added to a 110 × 27 mm glass tube, around which a horizontal line was drawn 80 mm above the bottom of the vial. When the experiment was begun, the number of flies that climbed above the mark on the vial after 10 s was recorded, and every trial was repeated 10 times. All behavioral studies were performed in a quiet isolation room at 25°C in 60–70% humidity under a red light. The climbing tested commenced on Day 3 after eclosion. After the climbing assay, the flies were maintained until death to calculate their lifespan.

### Spectrophotometric Determination of Intracellular Reduced Glutathione (GSH) and Malondialdehyde (MDA) Content

Drosophila were collected at DAY 36, ground on ice, and centrifuged at 12,000 × *g* for 6 min. The supernatant was taken, and the reaction was carried out according to the kit instructions. The intracellular GSH and MDA content was measured on a microplate reader.

### SH-SY5Y Cell Culture

SH-SY5Y cells were cultured in medium with equal amounts of MEM and F-12, supplemented with 1% nonessential amino acids (Gibco), 10% heat-inactivated fetal calf serum (FCS), 100 U/ml penicillin, and 100 U/ml streptomycin in a humid atmosphere of 5% CO2 and 95% air at 37°C. For routine cultures, cells were grown in dishes and passaged approximately twice per week when they reached confluence. Then, 5 nM rotenone was added to the culture every 3 days when the medium was changed. After 4 weeks, cells were treated with AA for 24 h or detected immediately without rotenone treatment. Solvents were used as parallel controls. For the acute model, cells were plated in 6-well plates at a density of approximately 3 × 10 (Rochet et al., 2004) viable cells. Twenty-four hours later, the cells were treated with AA (0.01–100 nM containing 0.1% DMSO, which had no toxic effect on the cells) for 24 h and then exposed to a fresh batch of the same medium containing 100 nM rotenone for 24 h.

### Preparation of Mouse Brain Mitochondria

Brain mitochondria were isolated from male ICR mice (weight 18–22 g) according to previous methods (Lai and Clark, 1979). Animal welfare and experimental procedures conformed to the Guide for the Care and Use of Laboratory Animals (Ministry of Science and Technology of China, 2006) and the related ethical regulations of our University. The experimental protocols were approved by ethics committee of Jiangsu University. Briefly, whole mouse brains, minus the cerebellum, were washed, minced, and homogenized in ice-cold isolation buffer (250 mmol/L sucrose, 10 mmol/L Tris–HCl, 0.5 mmol/L EDTA-K^+^, 0.1% BSA, pH 7.4). After different centrifugation, the pellet was suspended in 3% Ficoll medium (3% Ficoll, 250 mmol/L sucrose, 0.5 mmol/L EDTA-K^+^, and 10 mmol/L Tris–HCl, pH 7.4) and carefully layered onto 6% Ficoll medium. After being centrifuged at 11,500 × *g* for 30 min at 4°C, the mitochondrial pellet was suspended in isolation buffer (without BSA) and washed once. The final pellet was suspended in ice-cold storage buffer (250 mmol/L sucrose, 2.5 mmol/L KH_2_PO4, and 10 mmol/L Tris–HCl, pH 7.4). The protein concentration was determined by Bradford assay (Nanjing Jiancheng Bioengineering Institute, Nanjing, China). Fresh mitochondria were prepared for each experiment and used within 4 h after isolation.

### Fluorometric Analysis of Mitochondrial Membrane Potential (MMP)

Mitochondrial membrane potential reflects the functional state of the mitochondria within cells (Wadia et al., 1998). Changes in MMP were measured by the uptake of 5, 50, 6, 60-tetrachloro-1, 10, 3, 30-tetraethylbenzimidazolcarbocyanine iodide (JC-1) into the mitochondria. When excited at 488 nm, the monomeric form of JC-1 has an emission maximum at 525 nm, but the aggregated form (J-aggregates) has an emission maximum at 595 nm (Reers et al., 1991). Cells and isolated mitochondria were incubated with JC-1 at 37°C for 30 min in medium or reaction buffer (0.32 mmol/L sucrose, 10 mmol/L Tris, 20 mmol/L Mops, 50 μmol/L EGTA, 0.5 mmol/L MgCl_2_, 0.1 mmol/L Pi (K^+^), 5 mmol/L sodium succinate in the presence of 5 μg/mL JC-1). At the end of the incubation, the dye-loaded cells and mitochondria were collected by centrifugation, washed extensively with reaction buffer to remove excess dye, and then resuspended in the same buffer at the appropriate dilution. The cells were visualized under an inverted fluorescence microscope (Nikon, Ti-E Live Cell Imaging System Japan), and the fluorescence intensity was measured (488 nm excitation and 595 nm emission) on a Molecular Device spectrofluorometer (United States).

### Mitochondrial Mass

To count the mitochondria, a suspension of cells in free serum medium was loaded with 200 nmol/L MitoTracker Red FM for 30 min at 37°C. Fluorescence intensity was measured at an excitation wavelength of 581 nm and emission wavelength of 644 nm using a fluorescence spectrometer (Molecular Devices Corporation, Sunnyvale, CA, United States). Amounts were determined by comparing the intensity of the fluorescence signal that was produced by 1 × 10 (Rochet et al., 2004) cells.

### Measurement of ATP Synthesis by Mitochondria

ATP content was measured by luminometric assay, based on luciferin-luciferase reactions (Drew and Leeuwenburgh, 2003), using the Beyotime chemical luciferase ATP assay kit. Briefly, mitochondrial membranes were lysed in a buffer that contained 10 mmol/L Tris and 0.05% Triton X-100. Then, 50 μL of this lysate was added, and the luciferin-luciferase assay mixture was transferred to a white microplate. The results were measured on a multifunctional microplate reader (Perkinelmer, United States).

### Quantification of ROS Levels

Reactive oxygen species (ROS) levels were measured using the ROS-specific probe 5′,6′-chloromethyl-2′,7′-dichlorodihydrofluorescein diacetate (H2DCFDA, Beyotime Institute of Biotechnology, Nantong, China). Cells and mitochondria were incubated with 10 μmol/L H2-DCFDA for 30 min at 37°C in medium or reaction buffer (0.32 mmol/L sucrose, 10 mmol/L Tris, 20 mmol/L Mops, 50 μmol/L EGTA, 0.5 mmol/L MgCl_2_, 0.1 mmol/L Pi (K^+^), 5 mmol/L sodium succinate) (Friberg et al., 2002). Next, the cells were visualized under an inverted fluorescence microscope (Nikon, Ti-E Live Cell Imaging System, Japan), and the mitochondria were monitored kinetically for 60 min at 37°C on a Molecular Device spectrofluorometer (United States) with 488 nm excitation and 525 nm emission filters.

### Analysis of Mitochondrial Swelling

Mitochondrial swelling was assessed by measuring the absorbance of their suspension at 540 nm. Brain mitochondria were prepared in assay buffer (1 mg protein/mL) that contained 125 mmol/L sucrose, 50 mmol/L KCl, 2 mmol/L KH_2_PO_4_, 5 μmol/L rotenone, 10 mmol/L HEPES, and 5 mmol/L succinate. To induce mitochondrial swelling, 0.05 μg/μL α-syn was administered. Cyclosporine A (CsA) (50 nmol/L) was used as a positive reference (Elimadi et al., 2001). The extent of mitochondrial swelling was analyzed by measuring the decrease in absorbance (A) 0–60 min after the addition of α-syn at 37°C; a decrease in absorbance indicated an increase in mitochondrial swelling (Lee et al., 2002).

### Western Blot

Equal amounts of cell protein and mitochondria sample were loaded onto a 12% SDS-polyacrylamide gel, separated, and transferred to a nitrocellulose membrane, which was then incubated overnight with anti-Cyt C, anti-α-syn, or anti-PGC1-α. The secondary antibody (1:1000 dilution) was HRP-conjugated anti-mouse or anti-rabbit IgG (Boster Biological Technology, Wuhan, China). The signals were detected using ECL according to the manufacturer’s instructions and Kodak x-ray film.

### Statistical Analysis

Differences were tested by one-way analysis of variance (ANOVA), followed Student–Newman–Keuls test as a *post hoc* test. A value of *P* < 0.05 was considered significant. All experiments were done in triplicate and repeated three or four times.

## Results

### AA Improves Climbing Ability and Prolongs the Life Span in PD Drosophila

**FIGURE 1:**
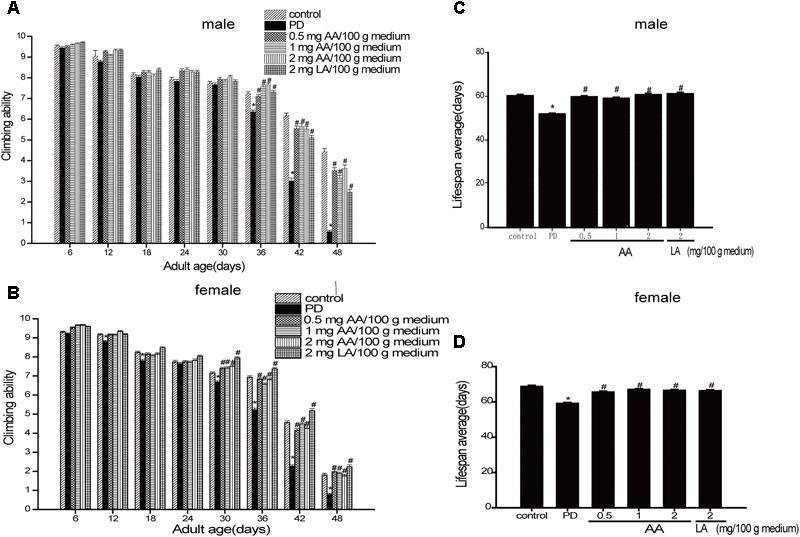
Neuroprotective effects of AA against α-syn-induced injury in α-syn transgenic PD drosophila. Control and a-syn transgenic PD Drosophila were divided into indicated groups, and the climbing ability and the number of dead drosophila were assayed on indicated day. AA promotes climbing in α-syn transgenic PD drosophila (*N* = 230; ^∗^*p* < 0.05 vs. non-PD group; ^#^*p* < 0.05 vs. PD group) for **(A)** male and **(C)** female Drosophila melanogaster (average). AA prolongs the life-span of α-syn transgenic PD drosophila in **(B)** males and **(D)** females (mean ± SEM, *N* = 230; ^∗^*p* < 0.05 vs. non-PD group; ^#^*p* < 0.05 vs. PD group)).

As shown in **Figures 1A,C** the climbing ability of all drosophila decreased progressively with age. However, PD drosophila (male and female) climbed more slowly than non-PD drosophila from Days 3 to 42. Notably, 0.5–2 mg AA/100 g of culture medium significantly improved the climbing response in male and female PD drosophila from 18 to 42 days. As shown in **Figures 1B,D** male and female transgenic PD drosophila lived longer than control drosophila. AA significantly extended the lifespan of PD drosophila.

### AA Has Antiapoptotic Effects in Rotenone-Induced SH-SY5Y Cell Damage

**FIGURE 2:**
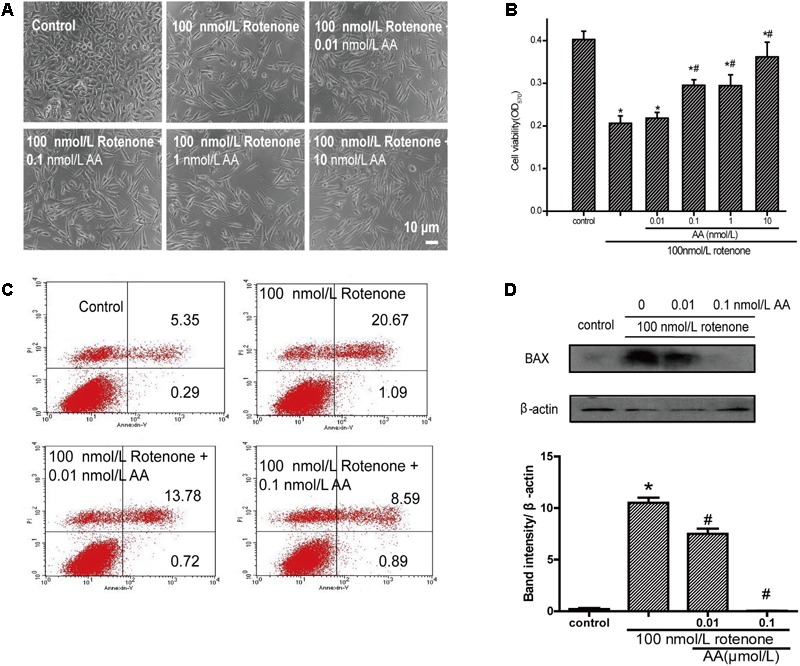
Anti-apoptotic effects of AA in rotenone-induced SH-SY5Y cell damage. **(A)** Morphology of SH-SY5Y cells after 24 h of treatment with various concentrations of AA after 24 h 100 nM rotenone exposure. Bar: 10 μm. **(B)** Cell viability of SH-SY5Y cells treated with AA after rotenone-induced cell damage. The data represent means ± SD *n* = 5. *P* < 0.05 vs. control group. **(C)** SH-SY5H cells were treated with AA and rotenone, and apoptosis rates of SH-SY5Y cells were measured by Annexin V/PI. **(D)** Translation levels of BAX were measured by western blot. Data are expressed as means ± SD, *n* = 3. ^∗^*p* < 0.01 vs. control group, ^#^*p* < 0.01 vs. rotenone group.

As shown in **Figure 2B**, the cell survival rate decreased after treatment with 100 nM rotenone. In contrast, 0.01–100 nM AA completely blocked the rotenone-induced decline in cell viability (**Figure 2B**). The pictures also show the same results compared with the rotenone group (**Figure 2A**), the treatment of AA (0.01–100 nM) prior to rotenone exposure clearly improved neuronal morphology, showing clear cell bodies and smooth processes. By flow cytometry, rotenone induced apoptosis in SH-SY5Y cells, which could be inhibited by AA (**Figure 2C**). The data on apoptotic protein–BAX also show the same results (**Figure 2D**).

### AA Has Antioxidative Effects in Parkinson-Like Injuries

**FIGURE 3:**
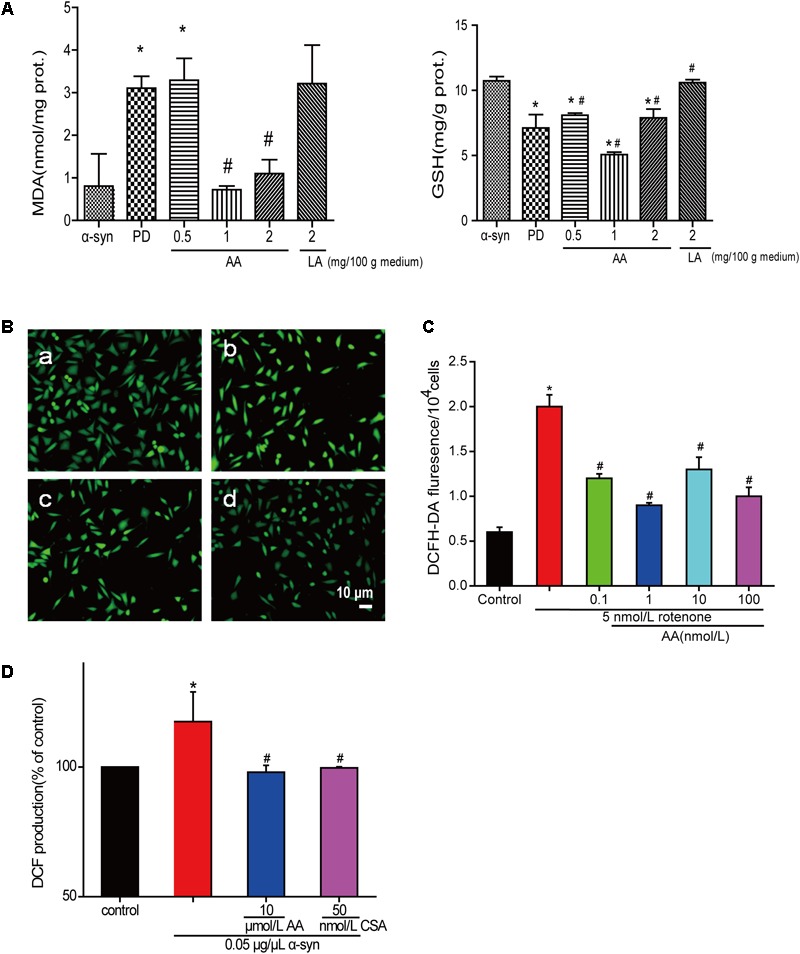
AA reverses PD-like oxidative stress. **(A)** Effects of AA on the activities of MDA and GSH in α-syn transgenic PD drosophila. Control and a-syn transgenic PD Drosophila were divided into indicated groups, and they all collected at DAY 36. The level of MDA and GSH in supernatant of drosophila homogenate were detected by kits. (mean ± SD, *n* = 3, ^∗^*p* < 0.05 vs. normal control group after treatment with different concentrations of AA or LA). **(B,C)** Intracellular ROS levels were determined based on DCF fluorescence under an inverted fluorescence microscope and on a spectrofluorometer. SH-SY5Y cells were treated with 5 nM rotenone exposure for 4 weeks and then various concentrations of AA after 24 h. a: control; b: 5 nmol/L chronic rotenone; c: 1 μM AA; d: 10 μM AA. Bar: 10 μm. Data are expressed as means ± SD, *n* = 3. ^∗^*p* < 0.01 vs. control group, #*p* < 0.01 vs. rotenone group. **(D)** Effects of AA on α-syn induced mitochondrial ROS formation (DCF production). Isolated mitochondria dyed with DCFH-DA were treated with vehicle or AA for 60 min at 37°C and exposed to 0 or 0.05 μg/μL α-syn at the same time. 60 min later fluorescence were detected on a spectrofluorometer. Data are expressed as means ± SD, *n* = 3. ^∗^*p* < 0.01 vs. control group, #*p* < 0.01 vs. α-syn group.

The amount of MDA often indicates the degree of lipid peroxidation in the body, indirectly reflecting the extent of cell damage. As shown in **Figure 3A**, MDA in PD flies increased by 2.8-fold compared with the normal control group, indicating a significant difference in the level of lipid peroxidation in drosophila. The MDA content in the AA group decreased from 64.5 to 76.7% compared with the PD group, showing a significant difference versus the control group (*P* < 0.01), indicating that AA antagonizes the overexpression of α-syn oxidative stress injury. However, 2 mg LA/100 g medium did not change the degree of lipid peroxidation in the fruit flies. The GSH content also showed the same results. It is indicated that AA could enhance the antioxidant capacity of PD fruit fly.

Asiatic acid also had antioxidative effects in SH-SY5Y cells and isolated brain mitochondria. As shown in **Figures 3B,C**, AA decreased the intensity of DCFH-DA fluorescence, which was elevated by rotenone. As seen in **Figure 3D**, untreated isolated mitochondria displayed substantial fluorescence, but mitochondria that were stressed with α-syn experienced an increase in ROS generation compared with untreated cells after 30 min. In contrast, 1–10 μmol AA/L attenuates the ROS generation that was induced by α-syn to normal levels. However, 100 μmol AA/L only partly attenuated the α-syn-induced ROS enhancement. CSA was a positive control and showed the same results as AA.

### AA Protects Mitochondria Against Rotenone- and α-Syn-Induced Injury in SH-SY5Y Cells and Isolated Mitochondria

**FIGURE 4:**
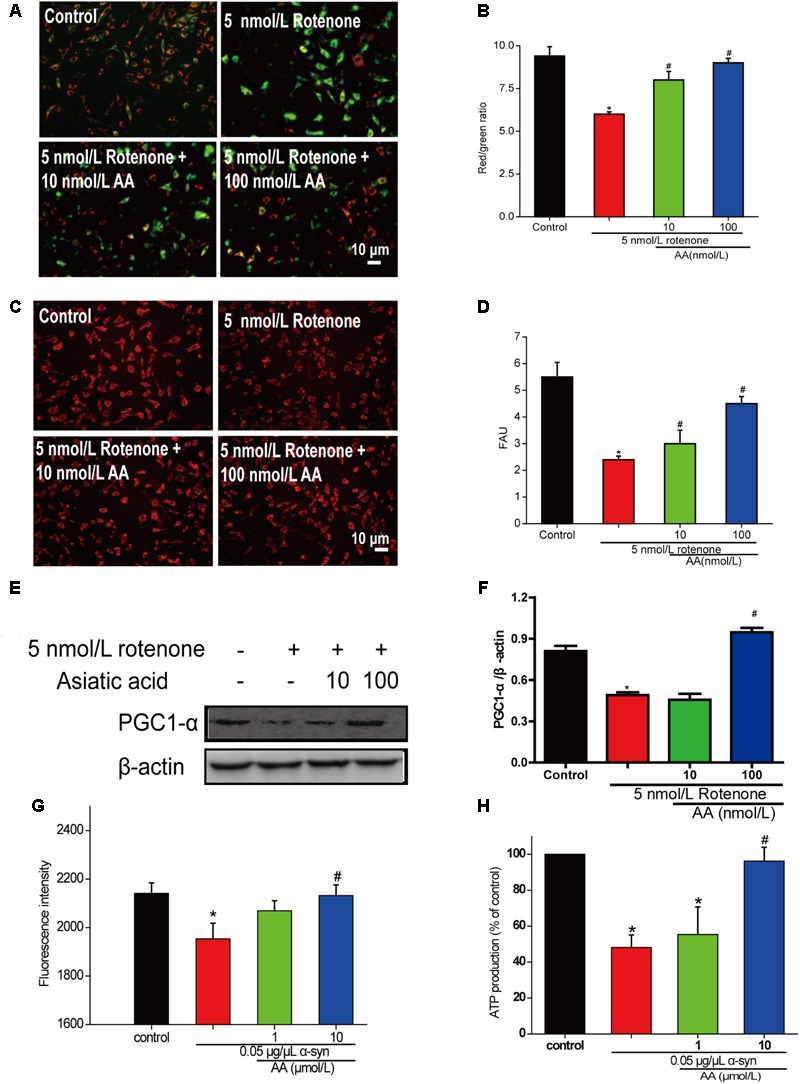
AA protects against mitochondrial dysfunction induced by PD-like injury. **(A,B)** SH-SY5Y cells were treated with 5 nM rotenone exposure for 4 weeks and then various concentrations of AA after 24 h. Intracellular red and green fluorescence of JC-1 was determined under an inverted fluorescence microscope **(A)** and on a spectrofluorometer **(B)**. Mitochondria number was determined with an inverted fluorescence microscope **(C)** and spectrofluorometer **(D)** and based on expression of PGC1-α **(E,H)**. **(F)** Effects of AA on mitochondrial membrane potential (JC-1 fluorescence intensity). Isolated mitochondria were treated with vehicle or AA for 60 min at 37°C and exposed to 0 or 0.05 μg/μL α-syn at the same time. Data are expressed as means ± SD, n = 3. ^∗^*p* < 0.01 vs. control group, ^#^*p* < 0.01 vs. rotenone group. **(G)** Effects of AA on mitochondrial ATP synthesis. Isolated mitochondria were treated with vehicle or AA for 60 min at 37°C and exposed to 0 or 0.05 μg/μL α-syn at the same time. The values are expressed as percentage of control, which is set to 100%. Data are expressed as means ± SD, *n* = 3. ^∗^*p* < 0.01 vs. control group, ^#^*p* < 0.01 vs. α-syn group. Bar: 10 μm.

To determine whether AA can protect mitochondrial function, we first measured MMP in cultured SH-SY5Y cells. From the fluorescence microscopy images, we found that rotenone induced a significant decrease in MMP in cultured SH-SY5Y (**Figures 4A,B**). Nevertheless, pretreatment with AA (1 and 10 nM) blocked this decline, which indicated that AA protects mitochondria (**Figures 4A,B**).

Next, we investigated the effects of AA against rotenone induced the number of mitochondria reduced in SH-SY5Y cells. As shown in **Figures 4C,D**, AA enhanced the red fluorescence intensity, reflecting an increase in the number of mitochondria. AA also elevated the level of PGC1-α (**Figures 4E,F**), indicating the biogenesis of mitochondria.

When mitochondria were exposed to 0.05 μg α-syn/μL for 60 min, a loss of mitochondrial membrane potential of approximately 10% of J-aggregate fluorescence was observed (**Figure 4G**). Co-incubation with AA (1–10 μmol/L) significantly prevented the α-syn-induced decline in JC-1 fluorescence intensity dose-dependently (**Figure 4G**). MMP is the driver of ATP synthesis, and its loss is expected to result in decreased ATP levels in cells and isolated mitochondria. As shown in **Figure 3B**, 0.05 μg α-syn/μL decreased ATP levels from 100 to 50% compared with control. Treatment with 1–10 μmol AA/L for 1 h effected significant ATP production compared with the α-syn group (**Figure 4H**).

### AA Lowers the Permeability of the Mitochondrial Membrane and Inhibits α-Syn Translocation to Mitochondria

**FIGURE 5:**
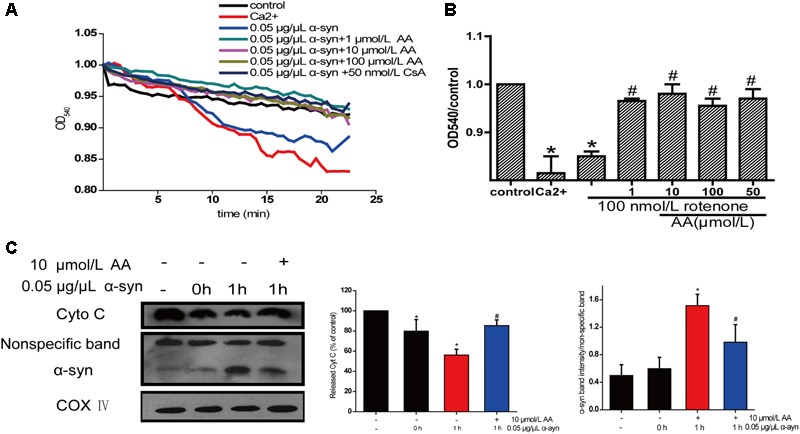
Neuroprotective effects of AA against α-syn-induced injury in isolated mitochondria. Isolated mitochondria were treated with vehicle or AA for 60 min at 37°C and exposed to 0 or 0.05 μg/μL α-syn at the same time. **(A,B)** Inhibitory effects of AA on α-syn-induced mitochondrial swelling, which was reflected by the decrease in absorbance at 540 nm and the results were statistics at the endpoint of the experiment. **(C)** AA reverses α-syn-induced release of cyt C from mitochondria and mitochondrial translocation of α-syn. Data are expressed as means ± SD, *n* = 3. ^∗^*p* < 0.01 vs. control group, #*p* < 0.01 vs. α-syn group.

The change in absorbance at 540 nm (A540) was measured to analyze mitochondrial swelling, which indirectly reflects the permeability of the mitochondrial membrane (Wills et al., 2010). The addition of 0.05 μg α-syn/μL to mitochondrial suspensions for 22.5 min resulted in a 6% decrease in A540, although the effect slightly lower than with 200 μmol/L Ca^2+^, which caused a 10% decrease compared with the control group. Mitochondrial swelling that was induced by α-syn was inhibited by pretreatment with 1–100 μmol AA/L for 3 min (**Figure 5A**)—an effect that was nearly the same as with CsA, a specific blocker of MPTPs, which lie in the sites where the mitochondrial inner and outer membranes meet. This result suggests that AA was.

The opening of MPTPs can stimulate the release of proapoptotic factors from the mitochondrial interspaces. Thus, we measured Cyt C in the mitochondria by immunoblotting. After mitochondria were incubated for 1 h with 0.05 μg α-syn/μL at 37°C, the level of Cyt C decreased markedly (**Figure 5B**), indicating its release into the extramitochondrial medium. Cotreatment with 10 μmol AA/L for 1 h partially inhibited α-syn-induced Cyt C release.

Next, we examined whether AA can block α-syn accumulation in the mitochondria. As shown in **Figure 5C**, after mitochondria were incubated for 1 h with 0.05 μg α-syn/μL at 37°C, α-syn amassed in the mitochondria, which could be inhibited by 10 μmol AA/L. These results indicate that AA blocks mitochondrial translocation of α-syn.

## Discussion

**FIGURE 6:**
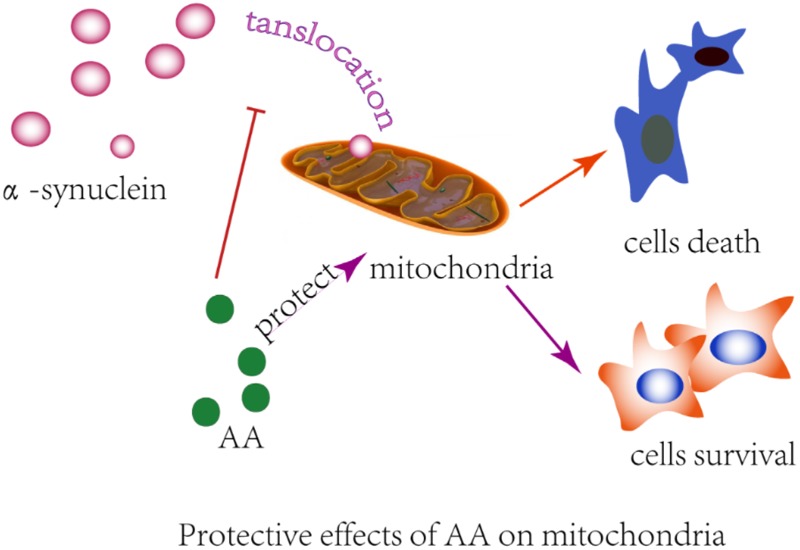
Illustration of the protective effects of AA in α-syn- and rotenone-induced PD-like models. AA may maintain the integrity of the mitochondrial membrane and then block α-syn importation into mitochondria.

Many observations suggest that α-syn causes neurodegeneration by interfering with multiple signaling pathways. α-syn protein can form plasma membrane channels or modify their activity, thus altering membrane permeability to ions; abnormally associate with mitochondria and cause mitochondrial dysfunction (e.g., mitochondrial depolarization, Ca^2+^ dyshomeostasis, cytochrome c release); and interfere with autophagy regulation (Ottolini et al., 2017). Previously, we found that AA protects neuronal cells against rotenone-induced mitochondrial dysfunctional injury (Xiong et al., 2009b). In the present study, we found that AA protects against rotenone- and α-syn-induced damage *in vivo* and *in vitro* through a mechanism that is related to directly preventing MPTPs from opening and inhibiting α-syn translocation to mitochondria (**Figure 6**). Our results are consistent with previous reports that in PD, α-syn aggregates are associated with intact mitochondria but interact with and cause nuclear degradation, which might be a major cause of cell death (Power et al., 2017).

We observed a significant improvement in the climbing response of PD flies with 0.5–2 mg AA/100 g of culture medium. These results suggest that AA exerts its neuroprotective effects through the prevention of oxidative damage via potent ROS-scavenging ability. AA extended the average lifespan of female PD flies at 0.5 mg/100 grams of culture medium.

Mitochondrial dysfunction has been implicated in the pathogenesis of many neurodegenerative diseases (Lin and Beal, 2006). In recent years, isolated mitochondria from brain and liver have been used frequently to identify the direct mechanisms of mitochondria in pathology and pharmacology (Jia et al., 2008; Gao et al., 2009; Banerjee et al., 2010), demonstrating that it is a good model for studying neurodegenerative diseases and screening drugs for neurodegenerative diseases.

Recent neurochemical studies in the postmortem brains of patients with PD have revealed significant increases in α-syn, indicating a relationship between the accumulation of α-syn and PD (Wills et al., 2010). Although the physiological functions of α-syn are unknown, more and more researchers have identified an association between α-syn and mitochondria in cellular and mouse models (Devi et al., 2008; Shavali et al., 2008; Liu et al., 2009). There is also substantial evidence that implicates α-syn and its ability to aggregate and bind vesicle membranes in the development of PD (Giannakis et al., 2008). α-syn can accumulate in mitochondria, based on its N-terminal 32-amino-acid region, which targets dopaminergic neurons and isolated mitochondria (Devi et al., 2008). In parallel, we found that additional α-syn could be imported to mitochondria in only 1 h—an effect that could be blocked by 10 μmol/L AA.

Mitochondrial import of α-syn is dependent on the mitochondrial transmembrane potential (ΔΨm) and mitochondrial ATP. Under our experimental conditions, AA attenuated the α-syn-induced MMP decline and ATP synthesis. This result is consistent with our previous study, in which AA rescued SH-SY5Y cells from rotenone- and H_2_O_2_-induced damage by preventing mitochondrial dysfunction (Xiong et al., 2009a). Several lines of evidence suggest that α-syn regulates membrane stability, neuronal plasticity, and enzymatic activity (Perez et al., 2002; Recchia, 2004; Bennett, 2005; Cookson, 2005; Shults, 2006). Moreover, constitutive levels of α-syn may be important for maintaining the functional integrity of the mitochondrial membrane (Ellis et al., 2005; Devi et al., 2008).

Impaired integrity of the mitochondrial membrane destroys the transmembrane proton gradient and interrupts the synthesis of ATP but also indirectly induces an increase in ROS and the loss of enzyme activity and can even trigger apoptosis (Norenberg and Rao, 2007). Consistent with these data, we observed ROS overproduction and the release of mitochondrial Cyt C—which can initiate apoptosis—due to disruptions in the integrity of the mitochondrial membrane structure by α-syn. However, this damage was reversed by AA, which might maintain the functional integrity of mitochondrial membranes.

Further, AA blocked α-syn-induced mitochondrial swelling in our study. Generally, mitochondrial swelling has been considered to result from an increase in inner membrane permeability and opening of MPTPs, high-conductance channels with several macromolecular components, including VDAC. AA regulates VDAC expression, according to our previous study (Xiong et al., 2009a), implying that the maintenance of the mitochondrial membrane by AA is due to preventing the MPTPs from opening.

In summary, this study has demonstrated the protective effects of AA in α-syn- and rotenone-induced PD-like models. We found that this protection occurs mainly through the maintenance of membrane integrity and blockade α-syn importation into mitochondria (**Figure 4**). We also found that AA reverses the increase in MDA levels and the decline in GSH content that is induced by α-syn overexpression in PD flies. AA reduces oxidative stress, against the α-syn aggregation caused by cell death, to protect nerve cells and reduce or even revert the symptoms of PD.

## Author Contributions

HD and YX designed and conducted the research and wrote the paper. JS and CC performed the statistical analysis. JG supervised the study. HX reviewed the paper. All authors have read and approved the final manuscript.

## Conflict of Interest Statement

The authors declare that the research was conducted in the absence of any commercial or financial relationships that could be construed as a potential conflict of interest.

## References

[B1] Banerjee K. Sinha M. Pham Cle L. Jana S. Chanda D. Cappai R. (2010). alpha-Synuclein induced membrane depolarization and loss of phosphorylation capacity of isolated rat brain mitochondria: implications in Parkinson’s disease. *FEBS Lett.* 584 1571–1576. 10.1016/j.febslet.2010.03.012 20226185

[B2] Bennett M. C. (2005). The role of alpha-synuclein in neurodegenerative diseases. *Pharmacol. Ther.* 105 311–331. 10.1016/j.pharmthera.2004.10.010 15737408

[B3] Cookson M. R. (2005). The biochemistry of Parkinson’s disease. *Annu. Rev. Biochem.* 74 29–52. 10.1146/annurev.biochem.74.082803.133400 15952880

[B4] Devi L. Raghavendran V. Prabhu B. M. Avadhani N. G. Anandatheerthavarada H. K. (2008). Mitochondrial import and accumulation of alpha-synuclein impair complex I in human dopaminergic neuronal cultures and Parkinson disease brain. *J. Biol. Chem.* 283 9089–9100. 10.1074/jbc.M710012200 18245082 PMC2431021

[B5] Drew B. Leeuwenburgh C. (2003). Method for measuring ATP production in isolated mitochondria: ATP production in brain and liver mitochondria of Fischer-344 rats with age and caloric restriction. *Am. J. Physiol. Regul. Integr. Comp. Physiol.* 285 R1259–R1267. 10.1152/ajpregu.00264.2003 12855419

[B6] Elimadi A. Sapena R. Settaf A. Le Louet H. Tillement J. Morin D. (2001). Attenuation of liver normothermic ischemia-reperfusion injury by preservation of mitochondrial functions with S-15176, a potent trimetazidine derivative1. *Biochem. Pharmacol.* 62 509–516. 10.1016/S0006-2952(01)00676-1 11448461

[B7] Ellis C. E. Murphy E. J. Mitchell D. C. Golovko M. Y. Scaglia F. Barceló-Coblijn G. C. (2005). Mitochondrial lipid abnormality and electron transport chain impairment in mice lackingα-synuclein. *Mol. Cell. Biol.* 25 10190–10201. 10.1128/MCB.25.22.10190-10201.2005 16260631 PMC1280279

[B8] Feany M. B. Bender W. W. (2000). A *Drosophila* model of Parkinson’s disease. *Nature* 404 394–398. 10.1038/35006074 10746727

[B9] Friberg H. Wieloch T. Castilho R. F. (2002). Mitochondrial oxidative stress after global brain ischemia in rats. *Neurosci. Lett.* 334 111–114. 10.1016/S0304-3940(02)01116-3 12435484

[B10] Gao X. Zheng C. Y. Yang L. Tang X. C. Zhang H. Y. (2009). Huperzine A protects isolated rat brain mitochondria against [beta]-amyloid peptide. *Free Radic. Biol. Med.* 46 1454–1462. 10.1016/j.freeradbiomed.2009.02.028 19272446

[B11] Giannakis E. Pacífico J. Smith D. P. Hung L. W. Masters C. L. Cappai R. (2008). Dimeric structures of α-synuclein bind preferentially to lipid membranes. *Biochem. Biophys. Acta* 1778 1112–1119. 10.1016/j.bbamem.2008.01.012 18261456

[B12] Hoogerheide D. P. Gurnev P. A. Rostovtseva T. K. Bezrukov S. M. (2017). Mechanism of alpha-synuclein translocation through a VDAC nanopore revealed by energy landscape modeling of escape time distributions. *Nanoscale* 9 183–192. 10.1039/c6nr08145b 27905618 PMC6298227

[B13] Jellinger K. (2012). The role of alpha-synuclein in neurodegeneration - An update. *Transl. Neurosci.* 3 75–122. 10.2478/s13380-012-0013-1

[B14] Jia H. Li X. Gao H. Feng Z. Li X. Zhao L. (2008). High doses of nicotinamide prevent oxidative mitochondrial dysfunction in a cellular model and improve motor deficit in a Drosophila model of Parkinson’s disease. *J. Neurosci. Res.* 86 2083–2090. 10.1002/jnr.21650 18381761

[B15] Krishnamurthy R. G. Senut M. C. Zemke D. Min J. Frenkel M. B. Greenberg E. J. (2009). Asiatic acid, a pentacyclic triterpene from *Centella asiatica*, is neuroprotective in a mouse model of focal cerebral ischemia. *J. Neurosci. Res.* 87 2541–2550. 10.1002/jnr.22071 19382233 PMC2941770

[B16] Lai J. C. Clark J. B. (1979). Preparation of synaptic and nonsynaptic mitochondria from mammalian brain. *Methods Enzymol.* 55 51–60. 10.1016/0076-6879(79)55008-3 459854

[B17] Lee C. S. Han J. H. Jang Y. Y. Song J. H. Han E. S. (2002). Differential effect of catecholamines and MPP^+^ on membrane permeability in brain mitochondria and cell viability in PC12 cells. *Neurochem. Int.* 40 361–369. 10.1016/S0197-0186(01)00069-9 11792467

[B18] Lee K. Y. Bae O. N. Serfozo K. Hejabian S. Moussa A. Reeves M. (2012). Asiatic acid attenuates infarct volume, mitochondrial dysfunction, and matrix metalloproteinase-9 induction after focal cerebral ischemia. *Stroke* 43 1632–1638. 10.1161/STROKEAHA.111.639427 22511009 PMC3361557

[B19] Lee M. K. Kim S. R. Sung S. H. Lim D. Kim H. Choi H. (2000). Asiatic acid derivatives protect cultured cortical neurons from glutamate-induced excitotoxicity. *Res. Commun. Mol. Pathol. Pharmacol.* 108 75–86. 11758977

[B20] Lee V. M. Y. Trojanowski J. Q. (2006). Mechanisms of Parkinson’s disease linked to pathological α-synuclein: new targets for drug discovery. *Neuron* 52 33–38. 10.1016/j.neuron.2006.09.026 17015225

[B21] Lin M. T. Beal M. F. (2006). Mitochondrial dysfunction and oxidative stress in neurodegenerative diseases. *Nature* 443 787–795. 10.1038/nature05292 17051205

[B22] Liu G. Zhang C. Yin J. Li X. Cheng F. Li Y. (2009). alpha-Synuclein is differentially expressed in mitochondria from different rat brain regions and dose-dependently down-regulates complex I activity. *Neurosci. Lett.* 454 187–192. 10.1016/j.neulet.2009.02.056 19429081

[B23] Long J. G. Gao H. X. Sun L. J. Liu J. K. Zhao-Wilson X. (2009). Grape extract protects mitochondria from oxidative damage and improves locomotor dysfunction and extends lifespan in a Drosophila Parkinson’s disease model. *Rejuvenation Res.* 12 321–331. 10.1089/rej.2009.0877 19929256

[B24] Mook-Jung I. Shin J. E. Yun S. H. Huh K. Koh J. Y. Park H. K. (1999). Protective effects of asiaticoside derivatives against beta-amyloid neurotoxicity. *J. Neurosci. Res.* 58 417–425. 10.1002/(SICI)1097-4547(19991101)58:3<417::AID-JNR7>3.0.CO;2-G 10518115

[B25] Moore D. J. West A. B. Dawson V. L. Dawson T. M. (2005). Molecular pathophysiology of Parkinson’s disease. *Annu. Rev. Neurosci.* 28 57–87. 10.1146/annurev.neuro.28.061604.135718 16022590

[B26] Norenberg M. D. Rao K. V. (2007). The mitochondrial permeability transition in neurologic disease. *Neurochem. Int.* 50 983–997. 10.1016/j.neuint.2007.02.008 17397969 PMC4714712

[B27] Ottolini D. Cali T. Szabo I. Brini M. (2017). Alpha-synuclein at the intracellular and the extracellular side: functional and dysfunctional implications. *Biol. Chem.* 398 77–100. 10.1515/hsz-2016-0201 27508962

[B28] Pendleton R. G. Parvez F. Sayed M. Hillman R. (2002). Effects of pharmacological agents upon a transgenic model of Parkinson’s disease in *Drosophila melanogaster*. *J. Pharmacol. Exp. Ther.* 300 91–96. 10.1124/jpet.300.1.91 11752102

[B29] Perez R. G. Waymire J. C. Lin E. Liu J. J. Guo F. Zigmond M. J. (2002). A role for alpha-synuclein in the regulation of dopamine biosynthesis. *J. Neurosci.* 22 3090–3099. 10.1523/JNEUROSCI.22-08-03090.2002 11943812 PMC6757524

[B30] Power J. H. T. Barnes O. L. Chegini F. (2017). Lewy bodies and the mechanisms of neuronal cell death in Parkinson’s disease and dementia with lewy bodies. *Brain Pathol.* 27 3–12. 10.1111/bpa.12344 26667592 PMC8029402

[B31] Recchia A. (2004). Alpha-Synuclein and Parkinson’s disease. *FASEB J.* 18 617–626. 10.1096/fj.03-0338rev 15054084

[B32] Reers M. Smith T. W. Chen L. B. (1991). J-aggregate formation of a carbocyanine as a quantitative fluorescent indicator of membrane potential. *Biochemistry* 30 4480–4486. 10.1021/bi00232a015 2021638

[B33] Rochet J. C. Outeiro T. F. Conway K. A. Ding T. T. Volles M. J. Lashuel H. A. (2004). Interactions among alpha-synuclein, dopamine, and biomembranes: some clues for understanding neurodegeneration in Parkinson’s disease. *J. Mol. Neurosci.* 23 23–34. 10.1385/JMN:23:1-2:023 15126689

[B34] Shavali S. Brown-Borg H. M. Ebadi M. Porter J. (2008). Mitochondrial localization of alpha-synuclein protein in alpha-synuclein overexpressing cells. *Neurosci. Lett.* 439 125–128. 10.1016/j.neulet.2008.05.005 18514418 PMC2502066

[B35] Sherer T. B. Betarbet R. Stout A. K. Lund S. Baptista M. Panov A. V. (2002). An in vitro model of Parkinson’s disease: linking mitochondrial impairment to altered alpha-synuclein metabolism and oxidative damage. *J. Neurosci.* 22 7006–7015. 10.1523/JNEUROSCI.22-16-07006.2002 12177198 PMC6757862

[B36] Shults C. W. (2006). Lewy bodies. *Proc. Natl. Acad. Sci. U.S.A.* 103 1661–1668. 10.1073/pnas.0509567103 16449387 PMC1413649

[B37] Spillantini M. G. Schmidt M. L. Virginia M.-Y. Lee J. Q. Trojanowski R. J. Michel G. (1997). α-Synuclein in Lewy bodies. *Nature* 388 839–840. 10.1038/42166 9278044

[B38] Wadia J. S. Chalmers-Redman R. M. Ju W. J. Carlile G. W. Phillips J. L. Fraser A. D. (1998). Mitochondrial membrane potential and nuclear changes in apoptosis caused by serum and nerve growth factor withdrawal: time course and modification by (-)-deprenyl. *J. Neurosci.* 18 932–947. 10.1523/JNEUROSCI.18-03-00932.1998 9437015 PMC6792769

[B39] Wills J. Jones J. Haggerty T. Duka V. Joyce J. N. Sidhu A. (2010). Elevated tauopathy and alpha-synuclein pathology in postmortem Parkinson’s disease brains with and without dementia. *Exp. Neurol.* 225 210–218. 10.1016/j.expneurol.2010.06.017 20599975 PMC2922478

[B40] Xiong Y. Ding H. Xu M. Gao J. (2009a). Protective effects of asiatic acid on rotenone-or H_2_O_2_-induced injury in SH-SY5Y cells. *Neurochem. Res.* 34 746–754. 10.1007/s11064-008-9844-0 18802751

[B41] Xiong Y. Y. Ding H. Q. Xu M. F. Gao J. (2009b). Protective effects of asiatic acid on rotenone- or H_2_O_2_-induced injury in SH-SY5Y cells. *Neurochem. Res.* 34 746–754. 10.1007/s11064-008-9844-0 18802751

[B42] Xu M. F. Xiong Y. Y. Liu J. K. Qian J. J. Zhu L. Gao J. (2012). Asiatic acid, a pentacyclic triterpene from *Centella asiatica*, is neuroprotective in a mouse model of focal cerebral ischemia. *Acta Pharmacol. Sin.* 87 578–587. 10.1038/aps.2012.3 22447225 PMC4010358

[B43] Zhang X. Wu J. Dou Y. Xia B. Rong W. Rimbach G. (2012). Asiatic acid protects primary neurons against C2-ceramide-induced apoptosis. *Eur. J. Pharmacol.* 679 51–59. 10.1016/j.ejphar.2012.01.006 22296759

